# Evaluation of fetal subarachnoid space using transabdominal ultrasonography and normal values during pregnancy

**DOI:** 10.1186/s40064-016-3121-5

**Published:** 2016-08-30

**Authors:** Aytul Corbacioglu Esmer, Atil Yuksel, Tugce Aksu Uzunhan, Omer Demir, Tugba Sarac Sivrikoz, Nur Aydinli

**Affiliations:** 1 Division of Maternal Fetal Medicine, Department of Obstetrics and Gynecology, Kanuni Sultan Suleyman Research and Teaching Hospital, Istanbul, Turkey; 2 Division of Maternal Fetal Medicine, Department of Obstetrics and Gynecology, Istanbul University Faculty of Medicine, Istanbul, Turkey; 3 Division of Pediatric Neurology, Department of Pediatrics, Istanbul University Faculty of Medicine, Istanbul, Turkey

**Keywords:** Subarachnoid space, Prenatal diagnosis, Ultrasound, Cranial scan, Nomogram

## Abstract

**Objectives:**

To determine the feasibility of evaluating the subarachnoid space by measuring two novel sonographic parameters in axial section using transabdominal ultrasound, in addition to the parameters previously defined in coronal section, and to construct a normal range for the subarachnoid space width in singleton healthy fetuses.

**Methods:**

Healthy pregnant women between 20 and 29 weeks were scanned using transabdominal ultrasound. Four variables were measured for the evaluation of subarachnoid space width; sinocortical width and anterior craniocortical width in coronal plane, and lateral and posterior craniocortical width in axial plane.

**Result:**

The data of 154 patients were recorded. SCW could be measured in 87.6 % (135) of fetuses, while the same figure was 77.9 % (120), 96.1 % (151) and 98.1 % (148) for anterior, lateral and posterolateral CCW, respectively. The SCW and anterior CCW did not display a significant correlation with gestational age and head circumference. The mean of SCW was 1.55 ± 0.41 mm with a range of 0.85–3.87 mm. The mean anterior CCW was 1.63 ± 0.39 mm with a range of 0.85–2.82 mm. A linear regression line was plotted between gestational age and lateral CCW (*r* = 0.707; *p* < 0.0001) and posterolateral CCW (*r* = 0.437; *p* < 0.0001), and nomograms for these parameters are constructed.

**Conclusion:**

This study presents a novel approach for the in utero evaluation of the subarachnoid space with two measurements in axial plane using transabdominal ultrasound. The nomograms will be helpful when there is a suspicion of subarachnoid space dilatation during routine cranial scan.

## Background

Dilatation of the subarachnoid space is mostly a benign condition in which cerebrospinal fluid accumulates transiently in frontal region in otherwise normal children with macrocephaly, and a spontaneous resolution is expected by 18–24 months of age as a result of progressive maturation of the structures of cerebrospinal fluid absorption (Paciorkowski and Greenstein 2007). On the other hand, it may be a sign of serious disorders such as lissencephaly and cortical atrophy, in microcephalic or normocephalic infants (Paciorkowski and Greenstein 2007; Ghai et al. 2006). Additionally, it is detected in several genetic conditions such as mucopolysaccharidosis, achondroplasy, Sotos syndrome and glutaricaciduria type I (Paciorkowski and Greenstein 2007).

Subarachnoid space width in infants has been evaluated by ultrasonography in several studies (Libicher and Tröger 1992; Lam et al. 2001; Frankel et al. 1998), but there is only one study which investigated the normal values in fetuses (Malinger et al. 2000). The authors measured sinocortical width (SCW) and craniocortical width (CCW) in coronal section using transvaginal ultrasound (Malinger et al. 2000). They concluded that dilatation of the subarachnoid space is an important finding which should alert obstetricians to possible intracranial pathologies necessitating a detailed neurosonography (Malinger 2000). Nevertheless, transvaginal approach is not a part of routine sonographic examination, and there is a need for a more practical method of imaging subarachnoid space in routine sonographic planes with transabdominal approach.

The purpose of this study was to determine the feasibility of evaluating the subarachnoid space by measuring two novel sonographic parameters in axial section using transabdominal ultrasound, in addition to the parameters previously defined in coronal section. We also aimed to construct a normal range for the subarachnoid space width in singleton healthy fetuses.

## Methods

This cross-sectional study conducted between December 2013 and May 2014 was approved by the Institutional Ethics Committee of Istanbul Faculty of Medicine. Informed consent was obtained from all participants. A total of 161 healthy pregnant women between 20 and 29 weeks based on the last menstrual period and/or first trimester sonographic examination were included in the study. All patients underwent detailed anatomic survey and fetal echocardiography in a standardized fashion using a transabdominal 2–7 MHz transducer (Voluson 730 Expert, GE Healthcare, Milwaukee, WI, USA). Multiple gestation, chromosomal or structural congenital anomalies, fetal macrosomia and intrauterine growth restriction constituted exclusion criteria.

**Fig. 1:**
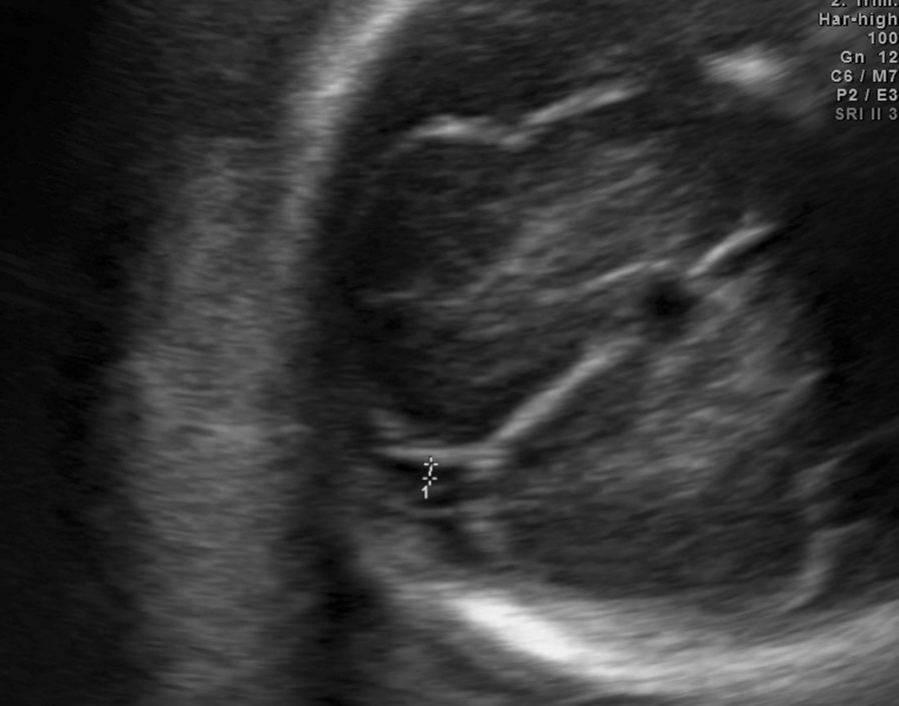
Sinocortical width is demonstrated in coronal plane

**Fig. 2:**
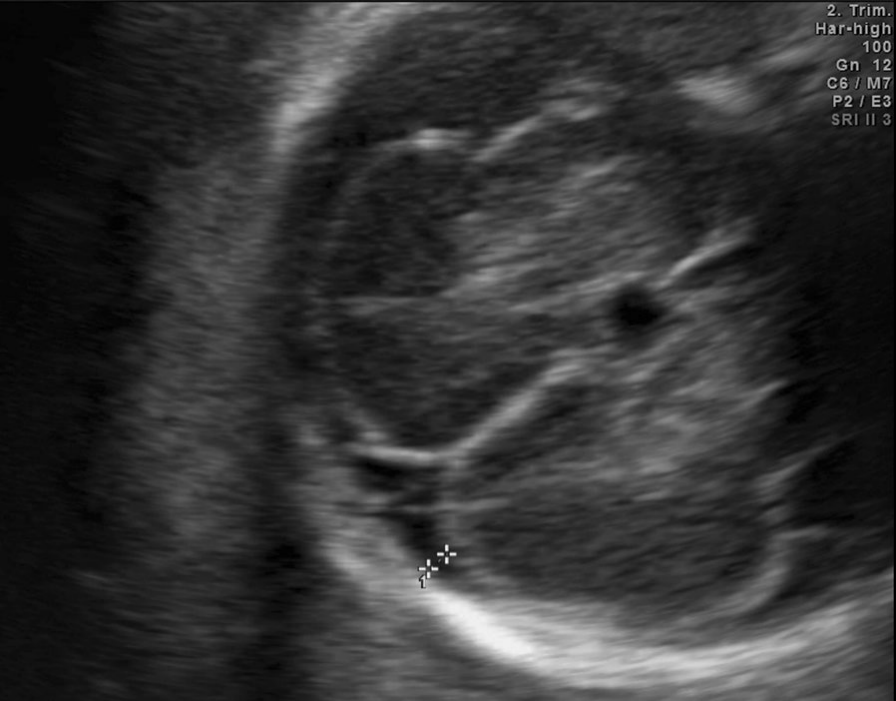
Anterior cranicortical width is demonstrated in coronal plane

**Fig. 3:**
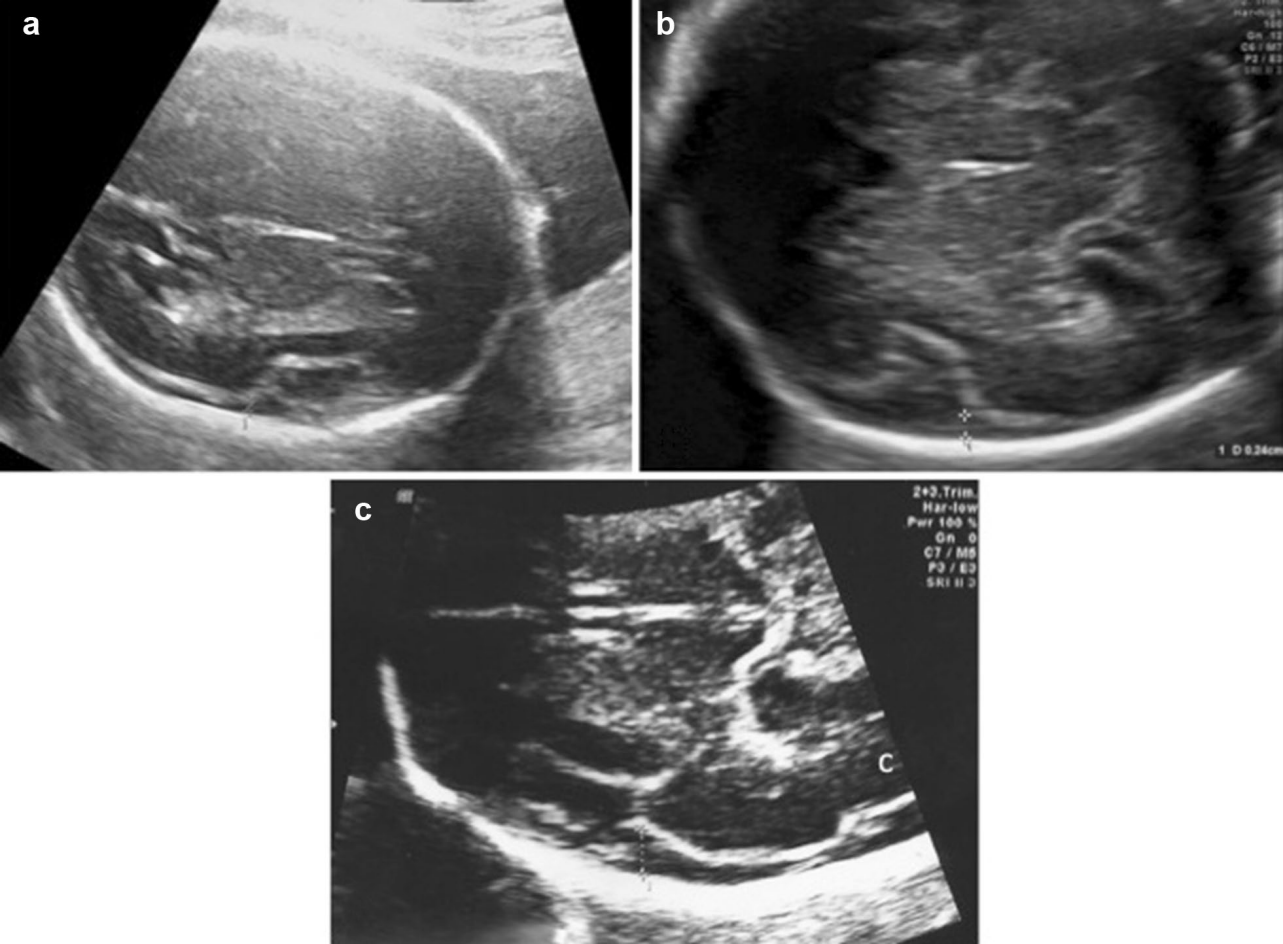
Lateral craniocortical width is demonstrated in axial plane at different gestational ages; 21 weeks’ gestation (**a**), 24 weeks’ gestation (**b**), 29 weeks’ gestation (**c**)

**Fig. 4:**
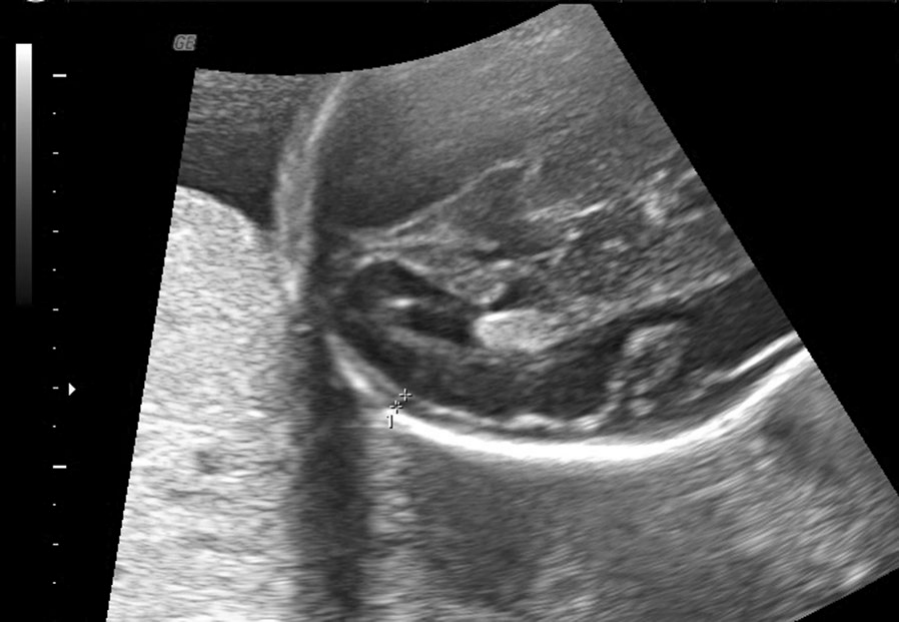
Posterolateral craniocortical width is demonstrated in axial plane

Four variables were measured for the evaluation of subarachnoid space width. Sinocortical width (SCW) and anterior craniocortical width (CCW) were evaluated in coronal plane at the level of the foramen of Monro as defined by Libicher and Tröger (1992). The former was defined as the shortest distance between the lateral wall of the triangular superior sagittal sinus and the surface of the adjacent cerebral cortex (Fig. 1), while the latter was the longest vertical distance between the calvarium and the surface of the cerebral cortex (Fig. 2). Only one side was measured, since differences between right and left sides were less than 1 mm in previous prenatal and postnatal studies Libicher and Tröger (1992), Malinger et al. (2000). In transventricular axial plane, the distal subarachnoid space width was measured perpendicular to midline at the level of posterior infolding margin of the slyvian fissure which forms first an obtuse angle and then an acute angle with the base as the gestation progresses (Fig. 3). This was called lateral CCW. The last measurement was performed also in transventricular axial plane at the level of the anterior margin of the lambdoid suture which was called posterolateral CCW (Fig. 4). All measurements were performed by the same physician (ACE). Each measurement was repeated three times for each fetus and the mean diameter was determined.

Head circumference (HC) and ventricular atrium measurements were recorded, and Spearman correlation test was performed to investigate a correlation between subarachnoid space width and these parameters, as well as the gestational age. Mann–Whitney *U* test was used to compare continuous variables. Intraobserver reliability was evaluated by means of intraclass correlation coefficient (ICC). All data processing was performed using the statistical software package SPSS 16.0 (SPSS, Chicago, IL, USA). Alpha significance level was accepted as 0.05 (p < 0.05).

## Results

A total of 161 singleton pregnant women were scanned. Seven cases were excluded from the study, because their postnatal data could not be reached. Therefore we analyzed the data of 154 fetuses. All of the fetuses were normal structurally. The mean maternal age, birth weight and gestational age at delivery were 30 ± 5.7 years, 3134 ± 597 g and 38.3 ± 2.2 weeks, respectively. We followed up the cases postnatally for 8–13 months. In two cases ventricular septal defect and in one case mild hypospadias was detected during the neonatal period. In another case posterior palate cleft was diagnosed postnatally. All cases had normal neurologic development.

SCW could be measured in 87.6 % (135) of fetuses, while the same figure was 77.9 % (120), 96.1 % (151) and 98.1 % (148) for anterior, lateral and posterolateral CCW, respectively. Intraobserver reproducibility was excellent with an ICC of 0.92 (95 % CI 0.9–0.94), 0.90 (95 % CI 0.87–0.93), 0.98 (95 % CI 0.97–0.98) and 0.95 (95 % CI 0.93–0.96) for SCW, anterior CCW, lateral CCW, and posterolateral CCW, respectively.

94 fetuses were female and 60 fetuses were male. The measurements did not show any significant differences between the two genders (p = 0.12, 0.66, 0.79 and 0.60 for SCW, anterior CCW, lateral CCW and posterolateral CCW, respectively).

**Fig. 5:**
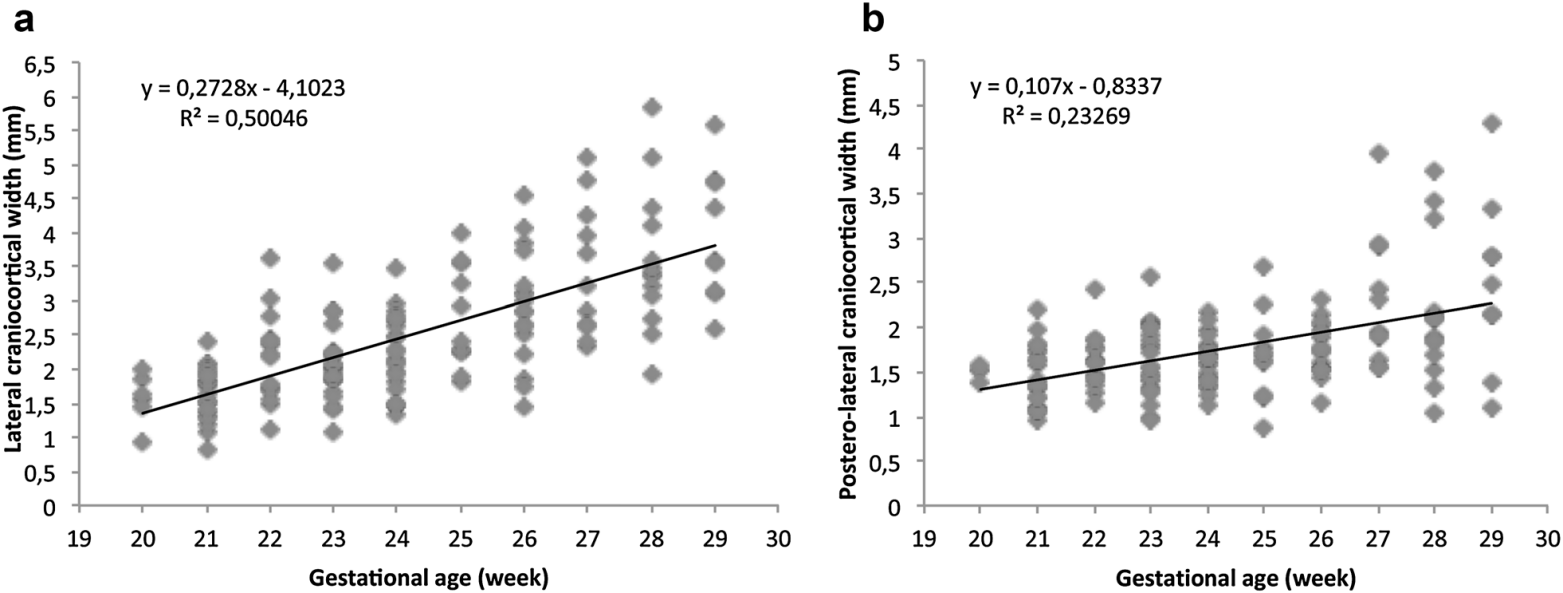
Relationship between gestational age and lateral CCW (**a**) and posterolateral CCW (**b**), and the linear regression line

**Fig. 6:**
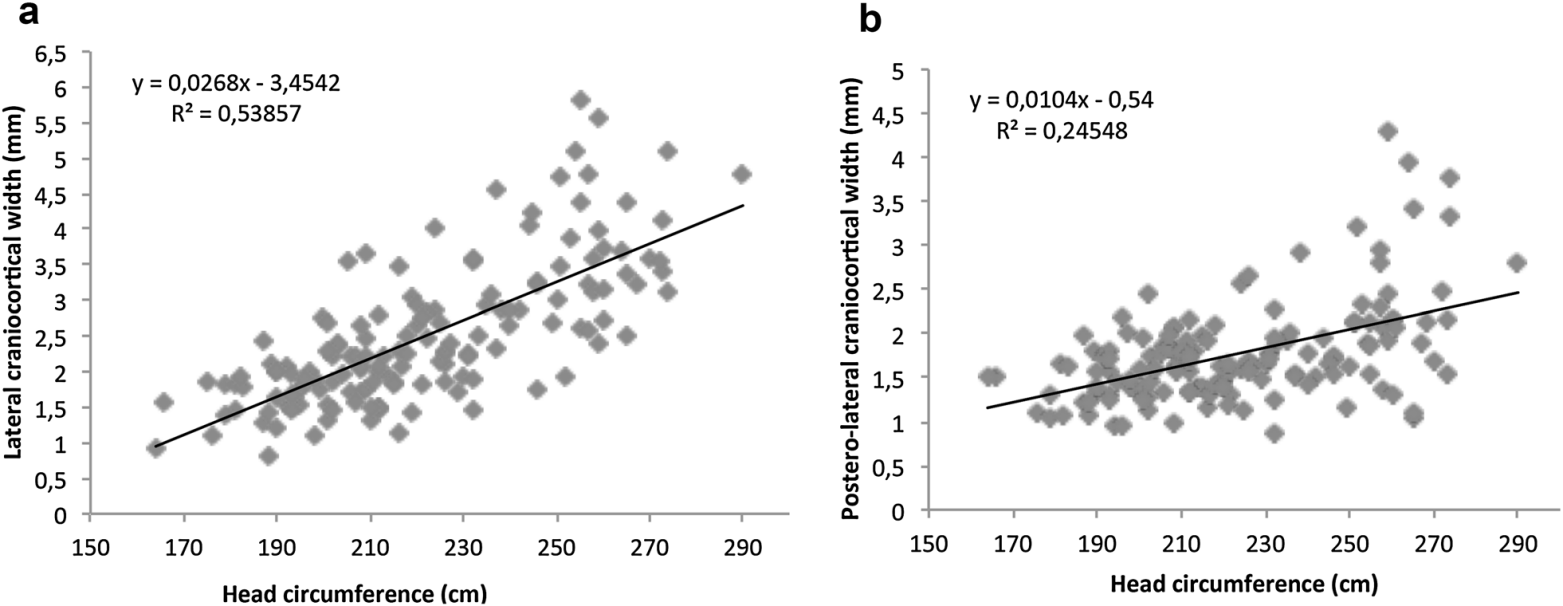
Relationship between head circumference and lateral CCW (**a**) and posterolateral CCW (**b**), and the linear regression line

The SCW and anterior CCW did not display a significant correlation with gestational age (*r* = 0.158 and 0.037; *p* = 0.08 and 0.69, respectively) and head circumference (*r* = 0.87 and −0.18; *p* = 0.32 and 0.85, respectively). Table 1 shows mean ± SD, range, 3rd, 10th, 50th, 90th and 97th percentile for SCW and anterior CCW. A linear regression line was plotted between gestational age and lateral CCW (*r* = 0.707; *p* < 0.0001; Fig. 5a) and posterolateral CCW (*r* = 0.437; *p* < 0.0001, Fig. 5b). A linear regression line was also plotted between head circumference, and lateral CCW (*r* = 0.709; *p* < 0.0001; Fig. 6a) and posterolateral CCW (*r* = 0.391; *p* < 0.0001; Fig. 6b). Nomograms for lateral and posterolateral CCW according to gestational age are shown in Tables 2 and 3. None of the subarachnoid measurements showed statistically significant correlation with ventricular atrial width.

**Table 1:** Sonographic measurements of the sinocortical and anterior craniocortical width (mm)

GA (week)	N	Mean ± SD	Min –max	3rd %ile	10th %ile	50th %ile	90th %ile	97th %ile
SCW	135	1.55 ± 0.41	0.85–3.87	0.99	1.14	1.49	2.10	2.49
Anterior CCW	120	1.63 ± 0.39	0.85–2.82	0.87	1.15	1.62	2.19	2.38

*SD* standard deviation, *Min* minimum, *Max* maximum, *SCW* sinocortical width, *CCW* craniocortical width

**Table 2:** Lateral craniocortical width (mm) according to gestational age

GA (week)	N	Mean ± SD	Min –max	3rd %ile	10th %ile	50th %ile	90th %ile	97th %ile
20–21	29	1.64 ± 0.37	0.81–2.42	0.81	1.09	1.64	2.06	2.42
22–23	39	2.10 ± 058	1.09–3.64	1.10	1.45	2.01	2.88	3.62
24–25	33	2.38 ± 0.67	1.33–4.00	1.33	1.49	2.27	3.51	3.99
26–27	28	3.12 ± 0.91	1.45–5.08	1.45	1.84	2.93	4.56	5.08
28–29	22	3.73 ± 1.01	1.94–5.82	1.94	2.54	3.51	5.42	5.82

*SD* standard deviation, *min* minimum, *max* maximum, *%ile* percentile

**Table 3:** Posterolateralcraniocortical width (mm) according to gestational age

GA (week)	N	Mean ± SD	Min –max	3rd %ile	10th %ile	50th %ile	90th %ile	97th %ile
20–21	25	1.46 ± 0.31	0.97–2.19	0.97	1.06	1.40	1.88	2.19
22–23	39	1.63 ± 0.36	0.95–2.57	0.96	1.19	1.61	2.88	2.55
24–25	32	1.64 ± 0.36	0.86–2.67	0.86	1.22	1.67	2.13	2.67
26–27	28	1.96 ± 0.57	1.15–3.95	1.15	1.51	1.89	2.91	3.95
28–29	24	2.28 ± 0.84	1.04–4.30	1.04	1.22	2.13	3.59	4.30

*SD* standard deviation, *min* minimum, *max* maximum, *%ile* percentile

The correlation between the four subarachnoid space width measurement parameters was analyzed. There was a statistically significant correlation between SCW and anterior CCW. Also, there was a significant correlation between posterolateral CCW and the other three parameters (Table 4).

**Table 4:** Correlation of the sonographic variables

Variable	*R* ^a^	*P*
SCW versus anterior CCW	0.477	<0.0001
SCW versus lateral CCW	0.190	0.831
SCW versus posterolateral CCW	0.250	0.004
Anterior CCW versus lateral CCW	0.061	0.515
Anterior CCW versus posterolateral CCW	0.221	0.017
Lateral CCW versus posterolateral CCW	0.428	<0.0001

*SCW* sinocortical width, *CCW* craniocortical width

^a^Spearman correlation coefficient

## Discussion

In postnatal studies SCW and CCW, which reflect the subarachnoid space in frontal region, were measured with transfontanel approach with a range of 0 and 6.3 mm (Libicher and Tröger 1992; Frankel et al. 1998; Narli et al. 2006; Sabouri et al. 2011). In another study, Libicher and Tröger suggested 3 and 4 mm as cut-off values for SCW and CCW, respectively, since these were the next integer above 95th percentile (Ghai et al. 2006). Similarly, in the only prenatal study, the investigators measured SCW and CCW with tranvaginal approach, and reported 1.4–5.8 and 1.5–6 mm as normal ranges (Malinger et al. 2000). In this study, we measured the same parameters in coronal section, but different from the previous study we measured two more parameters in axial section, which we referred to as lateral and posterolateral CCW. The rationale for this addition was the idea that axial rather than coronal planes are used in routine cranial scan. In this way, we managed to evaluate the subarachnoid space not only in frontal region but also in parietal region. Furthermore, unlike the previous study, we used transabdominal ultrasound in order to investigate its feasibility in evaluation of the subarachnoid space, since it is the method routinely used for sonographic screening examination.

The present study showed that all of the four measurements are reproducible and can be used to evaluate the pericerebral fluid accurately. However, visualizing SCW and anterior CCW in coronal section is not easy using tansabdominal ultrasound, and as this area cannot be seen clearly all the time the measurement might be inaccurate. On the other hand, measuring the subarachnoid space in axial section is easier and more feasible than coronal section, because in more than 95 % of cases the width was managed to be measured in this plane, while the same figure was 87.6 and 77.9 % for SCW and anterior CCW, respectively. As expected, there was an excellent correlation between SCW and anterior CCW, as well as between lateral and posterolateral CCW, because the former two represent the space in frontal region, whereas the latter two in parietal region. Furthermore, posterolateral CCW was weakly but statistically significantly correlated with both SCW and anterior CCW. For these reasons, it is reasonable to measure the subarachnoid space in axial plane instead of coronal plane during transabdominal scan.

We failed to show any significant relationship between gestational age, and SCW and anterior CCW. In consistent with our findings, the previous prenatal study did not show any relationship between SCW and gestational age (Malinger et al. 2000). However, the authors reported a slight but significant increase in the size of anterior CCW from 16 to 28 weeks, followed by a decrease until term (Malinger et al. 2000). Similarly, Frankel et al. (1998) examined 82 healthy newborns who were born at gestational age of 34.5–42 weeks, and reported a slightly negative linear relationship between the width of the subarachnoid space and the gestational age at birth. Moreover, unlike the measurements in coronal section, we demonstrated a linear relationship between gestational age and lateral CCW, as well as posterolateral CCW. We do not know the reason behind the difference between the coronal and axial measurement trends, but it might be due to different growth pattern of the cortex in frontal and parietal region.

Prenatal diagnosis of lissencephaly is very important due to the infavorable neurologic outcome, however, it is not possible to assess the sulcal development until late second trimester. Prominent subarachnoid space is a sonographic fetaure of Miller-Dieker syndrome, which is the most easily recognized form of lissencephaly on prenatal images, and it may be a warning sign in the earlier gestational age, as well as delayed sulcal development (Ghai et al. 2006). Furthermore, since there might also be a delay in sulcal development in ventriculomegaly, and ventriculomegaly may obscure visualization of the sulcal pattern of the parieto-occipital lobes, the presence or absence of prominent subarachnoid space is useful in differentiating isolated ventriculomegaly from lissencephaly (Fong et al. 2004). We believe that the assessment of pericerebral fluid in axial plane is more useful and practical, because it is readily visible when evaluating the lateral ventricules and insula. Moreover, benign enlargement of subarachnoid space is characterized by bifrontal widening, while brain atrophy is associated with a generalized dilatation (Maytal et al. 1987). This also supports the importance of evaluating the space in different areas and increases the worth of measurements in axial plane.

It was suggested that the measurement of subarachnoid space width is an indirect method of assessing and monitoring brain growth (Okur et al. 2013; Armstrong et al. 2002). In this study, we reported the normal ranges of the subarachnoid space width according to gestational age between 20 and 29 weeks of gestation. However, the prognosis of the otherwise normal fetuses with a dilated subarachnoid space is yet to be known. Malinger et al. (2000) reported three fetuses with isolated subarachnoid space dilatation; postnatally one of them was diagnosed to be trisomy 21 with brain atrophy, while the other two were associated with familial macrocephaly with favorable neurologic outcome. We believe that more studies are required for the adequate management and prenatal counseling of the cases with *in utero* dilatation of subarachnoid space.

In the present study, initially we planned to measure the subarachnoid space width between 16 and 38 weeks’ gestation, however, we managed to measure only between 20 and 29 weeks, because measuring the space is technically very difficult using transabdominal ultrasound before and after this period. However, cranial anatomy is mostly assessed within these gestational weeks, and the nomograms we constructed will provide valuable information for the diagnosis of several cranial pathologies.

In conclusion, this study presents a novel approach for the in utero evaluation of the subarachnoid space with two measurements in axial plane using transabdominal ultrasound. Furthermore, it provides data on the normal range of the subarachnoid space in fetuses. Although we do not advocate measurement of the subarachnoid space as a part of the routine anatomical survey, we believe that the nomograms will be helpful when there is a suspicion of subarachnoid space dilatation during routine cranial scan.
